# Biostimulation in primiparous postpartum acyclic early weaned beef cows: introducing the bulls at weaning advances cyclic rebreeding

**DOI:** 10.21451/1984-3143-AR2017-0051

**Published:** 2018-12-05

**Authors:** Rodolfo Ungerfeld

**Affiliations:** Departamento de Fisiología, Facultad de Veterinaria, Universidad de la República, Montevideo, Uruguay.

**Keywords:** anestrus, cattle, ovulation, socio-sexual stimulus, suckling

## Abstract

Weaning and biostimulation promote an early cyclic postpartum rebreeding. Although the signals and mechanisms by which weaning and biostimulation differ, both end stimulating LH secretion. The aim of the experiment was to determine if weaning and biostimulation have additive effects advancing the postpartum rebreeding in primiparous postpartum anoestrous cows. The experiment was performed during late spring – early summer with 51 primiparous Hereford cows. Six weeks after parturition calves were weaned, and cows were managed in two experimental groups: WB (n = 22) and WDB (n = 29). Bulls were joined with WB cows at weaning, but joining was delayed one week in WDB cows. The presence of corpora lutea in the ovaries was recorded weekly with ultrasound, and 28 and 50 days after the end of the exposure period, pregnancy was determined by ultrasound. The percentage of cyclic cows was greater in WB than in WDB on Weeks 8 (36.4 vs 0%), 9 68.2 vs 13.8%), 10 (86.4 vs 27.6%) and 11 (100.0 vs 37.9%) (P < 0.001 in all). Cows that were weaned and biostimulated simultaneously rebred earlier than WDB cows (Week 9.1 ± 0.2 vs Week 11.0 ± 0.2; P < 0.0001). At the end of the experiment 46/51 (90.2%) of the animals were cycling. Pregnancy rate tended to be greater in WB than WDB cows 28 days after the end of the breeding period 18/22 vs 17/29, P = 0.077), but there was no difference at the end of the study (20/22 vs 24/29, ns). In conclusion, the application of weaning and biostimulation simultaneously advances postpartum rebreeding more than weaning alone in beef cows.

## Introduction

Lactation has negative effects on cyclic rebreeding in postpartum cows as it increases the energetic requirements. However, suckling also has a direct effect inhibiting postpartum rebreeding: early weaned cows come into estrus and ovulate earlier than nursed cows ( Acosta *et al*., 1983 ; Quintans *et al*., 2009 ). This inhibition is mediated by the negative effect that suckling has in the secretion of LH pulses ( Stagg *et al*., 1998 ). In this sense, weaning triggers a rapid increase in LH secretion ( Stagg *et al*., 1998 ). Thus, weaning has also positive effects in the response to estrous synchronization treatments ( Geary *et al*., 2001 ). Therefore, different weaning strategies have been developed to increase LH secretion, and thus, advance postpartum rebreeding.

Another strategy to stimulate an advancement of postpartum rebreeding is biostimulation (see review: Fiol and Ungerfeld, 2011 ). In many studies it has been reported that exposure to males stimulates cyclic activity in postpartum cows ( Zalesky *et al*., 1984 ; Alberio *et al*., 1987 ; Landaeta-Hernández *et al*., 2004 ; Berardinelli and Joshi, 2005 ; Landaeta-Hernández *et al*., 2006 ). The introduction of bulls, or even androgenized steers stimulates an increase in LH pulse secretion in postpartum cows ( Fernandez *et al*., 1996 ; Tauck *et al*., 2010 ) and anestrous heifers ( Fiol and Ungerfeld, 2016 ).

Therefore, both, weaning and biostimulation promote an early cyclic postpartum rebreeding. Although the signals and mechanisms by which weaning and biostimulation differ (see reviews, for postpartum: Williams *et al*., 1996 ; for biostimulation: Fiol and Ungerfeld, 2011 ), both end stimulating LH secretion. In this sense, Silva Filho *et al*. (2016) reported that the combination of biostimulation and temporary weaning decreased the number of services required per conception and increased the pregnancy rate achieved in beef cows compared to biostimulation itself after an estrous synchronization treatment. However, it is not known if, as both practices end triggering the same endocrine response, the simultaneous application of weaning and biostimulation have synergistic effects. Therefore, the aim of the experiment was to determine if weaning and biostimulation have additive effects advancing the postpartum rebreeding in primiparous postpartum anoestrous cows.

## Methods

### Animals and location

The experiment was performed in a commercial farm located in Soriano, Uruguay, (33º S) between November and January (late spring – early summer) with 51 primiparous Hereford cows. Six weeks after parturition calves were weaned, and cows were managed in two experimental groups: WB (n = 22) and WDB (n = 29) (body condition = 2.79 ± 0.09; scale 1 to 8). Bulls were joined with WB cows at weaning, but joining was delayed one week in WDB cows. Body condition score (scale of one to eight: one = extremely emaciated, eight = excessively fat) was evaluated at the beginning and at the end of the experiment by the same observer.

Four 4-6-yr-old Angus bulls were selected according to a breeding soundness evaluation performed one month before beginning the experiment. Evaluation included a general physical examination and a particular reproductive exam of testicles and epydidimus.

### Ultrasonographic evaluation

The presence of corpora lutea in the ovaries was recorded weekly with ultrasound, using a Chison 500 Vet machine with a 7.5 MHz linear probe (Chison Medical Imaging, Wuxi, China). Ultrasound scanning began one week before weaning and continued 6 weeks more after weaning. Resumption of cyclic activity was considered as the first date that a corpus luteum was observed if it was observed in two successive scans in the same ovary. Twenty-eight and fifty days after the end of the exposure period, pregnancy was determined by ultrasound.

### Statistical analyses

The accumulated frequencies of cows with corpus luteum per week, and the final pregnancy rates were compared using chi square tests. Intervals to resumption of cyclic activity were compared with ANOVA and are presented as mean ± SEM. Birth was considered as Week 0.

## Results

**Figure 1:**
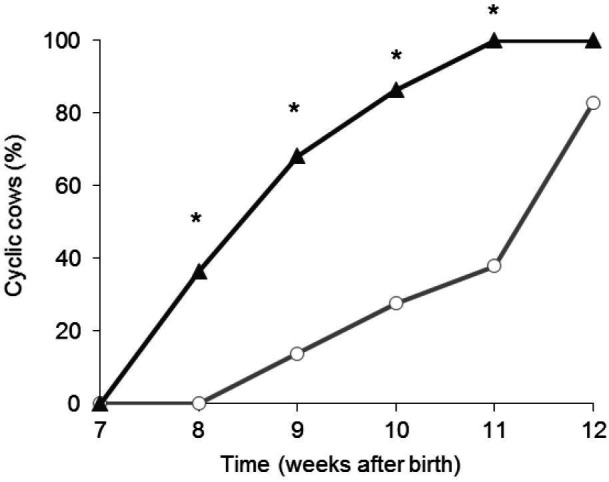
Percentage of cyclic cows after weaning (performed on Week 6), and joined with bulls on Week 6 (- ▲-, n=22) or Week 7 (- ○ -, n=29). All were primiparous Hereford cows.

No ovulation was detected until Week 8 (second week after weaning). However, at the end of the experiment 46/51 (90.2%) of the animals were cycling. The percentage of cyclic cows was greater (P < 0.001) in WB than in WDB on Weeks 8, 9, 10 and 11 ( Fig. 1 ). Cows that were weaned and biostimulated simultaneously (WB cows) rebreed earlier than WDB cows (Week 9.1 ± 0.2 vs Week 11.0 ± 0.2; P < 0.0001).

Pregnancy rate tended to be greater in WB tan WDB cows 28 days after the end of the breeding period 18/22 vs 17/29, P=0.077), but there was no difference at the end of the study (20/22 vs 24/29, ns).

## Discussion

Biostimulation applied simultaneously to weaning increased the proportion of cows that ovulated, as ovulation was delayed in the cows biostimulated one week later. Therefore, both practices can be applied simultaneously to advance rebreeding even more than with each of the practices alone. Although it has been clearly demonstrated that weaning is the strongest stimulation for rebreeding ( Williams *et al*., 1996 ), the simultaneous introduction of bulls potentiates the response to early weaning. From a practical view, these results open interesting possibilities for natural breeding in “clean” systems ( Martin and Kadokawa, 2006 ).

Although weaning and biostimulation probably triggered an increase in LH pulsatility, the simultaneous application of both practices advanced more the first ovulation that each one alone. This means that each is not triggering the maximum response or that the response is greater because both act through different mechanisms. Introduction of bulls induces an increase of LH ( Fiol and Ungerfeld, 2016 ), as happens in anoestrous sheep and goats ( Delgadillo *et al*., 2009 ). As suckling increases the synthesis of endogenous opioid (that inhibit GnRH secretion) and the central negative sensibility to oestrogens, weaning removes this inhibition. Therefore, as both stimulus advanced the response, it is probably that each one alone could not trigger the maximum increase in LH secretion.

In this study, cows were weaned and biostimulated shortly after birth, but most commonly, these managements are applied at later postpartum stages. In this sense, the possible advantages of including these managements may be even greater, as the GnRH and LH secretions are probably more sensitive for responding to external stimuli at later postpartum periods.

In conclusion, the application of weaning and biostimulation simultaneously advanced postpartum rebreeding more than weaning alone in primiparous Hereford cows.
